# TBCRC 002: a phase II, randomized, open-label trial of preoperative letrozole with or without bevacizumab in postmenopausal women with newly diagnosed stage 2/3 hormone receptor-positive and HER2-negative breast cancer

**DOI:** 10.1186/s13058-020-01258-x

**Published:** 2020-02-18

**Authors:** Christos Vaklavas, Brian S. Roberts, Katherine E. Varley, Nancy U. Lin, Minetta C. Liu, Hope S. Rugo, Shannon Puhalla, Rita Nanda, Anna Maria Storniolo, Lisa A. Carey, Mansoor N. Saleh, Yufeng Li, Jennifer F. Delossantos, William E. Grizzle, Albert F. LoBuglio, Richard M. Myers, Andres Forero-Torres, on behalf of the Translational Breast Cancer Research Consortium (TBCRC)

**Affiliations:** 1 University of Alabama at Birmingham, Birmingham, AL USA; 2 Present Address: Huntsman Cancer Institute, University of Utah, Salt Lake City, USA; 3 HudsonAlpha, Institute for Biotechnology, Huntsville, AL USA; 4 Dana-Farber Cancer Institute, Boston, MA USA; 5 Lombardi Cancer Center, Georgetown University Hospital, Washington, DC, USA; 6 University of California, San Francisco, USA; 7 Hellen Diller Family Comprehensive Cancer Center, San Francisco, CA USA; 8 University of Pittsburgh Medical Center, Magee Women’s Cancer Program, Pittsburgh, PA USA; 9 University of Chicago, Chicago, IL USA; 10 Melvin and Bren Simon Cancer Center at Indiana University, Indianapolis, IN USA; 11 University of North Carolina Lineberger Comprehensive Cancer Center, Chapel Hill, NC USA; 12 Georgia Cancer Specialists PC, Atlanta, GA USA

**Keywords:** Preoperative therapy, Bevacizumab, Letrozole, Breast cancer, Hormone receptor-positive breast cancer, Luminal breast cancer, Postmenopausal

## Abstract

**Background:**

In preclinical studies, the expression of vascular endothelial growth factor (VEGF) in hormone receptor-positive breast cancer is associated with estrogen-independent tumor growth and resistance to endocrine therapies. This study investigated whether the addition of bevacizumab, a monoclonal antibody against VEGF, to letrozole enhanced the antitumor activity of the letrozole in the preoperative setting.

**Methods:**

Postmenopausal women with newly diagnosed stage 2 or 3 estrogen and/or progesterone receptor-positive, HER2-negative breast cancer were randomly assigned (2:1) between letrozole 2.5 mg PO daily plus bevacizumab 15 mg/kg IV every 3 weeks (Let/Bev) and letrozole 2.5 mg PO daily (Let) for 24 weeks prior to definitive surgery. Primary objective was within-arm pathologic complete remission (pCR) rate. Secondary objectives were safety, objective response, and downstaging rate.

**Results:**

Seventy-five patients were randomized (Let/Bev *n* = 50, Let *n* = 25). Of the 45 patients evaluable for pathological response in the Let/Bev arm, 5 (11%; 95% CI, 3.7–24.1%) achieved pCR and 4 (9%; 95% CI, 2.5–21.2%) had microscopic residual disease; no pCRs or microscopic residual disease was seen in the Let arm (0%; 95% CI, 0–14.2%). The rates of downstaging were 44.4% (95% CI, 29.6–60.0%) and 37.5% (95% CI, 18.8–59.4%) in the Let/Bev and Let arms, respectively. Adverse events typically associated with letrozole (hot flashes, arthralgias, fatigue, myalgias) occurred in similar frequencies in the two arms. Hypertension, headache, and proteinuria were seen exclusively in the Let/Bev arm. The rates of grade 3 and 4 adverse events and discontinuation due to adverse events were 18% vs 8% and 16% vs none in the Let/Bev and Let arms, respectively. A small RNA-based classifier predictive of response to preoperative Let/Bev was developed and confirmed on an independent cohort.

**Conclusion:**

In the preoperative setting, the addition of bevacizumab to letrozole was associated with a pCR rate of 11%; no pCR was seen with letrozole alone. There was additive toxicity with the incorporation of bevacizumab. Responses to Let/Bev can be predicted from the levels of 5 small RNAs in a pretreatment biopsy.

**Trial registration:**

This trial is registered with ClinicalTrials.gov (Identifier:  NCT00161291), first posted on September 12, 2005, and is completed.

## Background

Angiogenesis is an integral step in tumor progression [1]. Specifically in hormone receptor-positive breast cancer, enhanced angiogenesis accelerated tumor growth [2], and overexpression of the vascular endothelial growth factor (VEGF) either by the tumor [3, 4] or stromal cells [5] was associated with hormone-independent growth and resistance to endocrine therapies. An interplay between estrogens and angiogenic activity exists [6, 7], and *VEGF* is one of the genes whose expression is modulated by estrogens. Indeed, the presence of functional estrogen response elements in the *VEGF* gene [8–10] underpins the transcriptional activation of *VEGF* by estrogens [11]. Tumor-released VEGF recruits stromal cells and promotes a desmoplastic microenvironment; stromal cells, in turn, provide mitogenic and angiogenic growth factors stimulating both tumor and stromal cell growth [4]. VEGF secreted by stromal cells and acting cooperatively with other factors can substitute for estrogens and foster hormone-independent growth of luminal tumors [5]. At the clinical level, in hormone receptor-positive breast cancer, elevated intratumoral levels of VEGF have been associated with suboptimal responses to hormonal therapies and poorer clinical outcomes [12–14] lending support to the hypothesis that VEGF and angiogenesis may contribute to resistance to endocrine therapies.

These preclinical studies set the stage for a pilot, single-institution, single-arm study of preoperative letrozole in combination with bevacizumab in postmenopausal women with hormone receptor-positive breast cancer [15]. In that study (*n* = 25), preoperative letrozole and bevacizumab for 24 weeks resulted in a pCR rate of 12% (*n* = 3), while the overall objective response rate was 68% (*n* = 17). The rate of downstaging to stage 0 or stage 1 was 32% [15]. The treatment was overall well tolerated with an 8% discontinuation rate (*n* = 2) due to adverse events.

The purpose of the current study was to evaluate the benefit of neoadjuvant/preoperative bevacizumab in combination with letrozole in postmenopausal women with hormone receptor-positive breast cancer. The principal hypothesis of the study was that concurrent inhibition of estrogen receptor (ER)-mediated signaling and VEGF-mediated stromal activation and angiogenesis would be more effective than ER blockade alone. The primary objective of the study was to determine the pCR rate with neoadjuvant letrozole for 24 weeks with or without bevacizumab; secondary objectives included assessments of toxicity, overall objective response, and downstaging rates in the two arms. The utility of circulating tumor and endothelial cells as biomarkers of response and pretreatment tumor genomic profiles associated with response or resistance to protocol therapy were explored.

## Methods

### Study design

This was a randomized selection (“pick-the-winner”), open-label, two-treatment arm, phase II study conducted through the Translational Breast Cancer Research Consortium across nine cancer centers in the USA. Bevacizumab was provided by Genentech. This study is registered on the clinical trial website of the US National Cancer Institute (http://www.clinicaltrials.gov/ct2/show/NCT00161291).

### Patient population

Eligible patients were postmenopausal women, 50 years or older, with newly diagnosed and pathologically confirmed invasive stage 2 and 3 breast cancer (T2, T3, T4a-c, N0-2, and M0), ER- and/or PR-positive, Her2/neu-negative (0 or 1 by immunohistochemistry or non-amplified by in situ hybridization; a 2+ score by immunohistochemistry had to be confirmed as non-amplified by in situ hybridization) who were candidates for mastectomy or breast conserving surgery. Eligible patients had to have measurable disease by mammogram and/or ultrasound; in special cases, dedicated breast magnetic resonance imaging (MRI) could be used. Patients were required to demonstrate controlled blood pressure (< 150/90 mmHg) and a normal ejection fraction with a multigated acquisition (MUGA) scan or echocardiogram at baseline.

Patients with inflammatory (T4d) or metastatic breast cancer were excluded. Other exclusion criteria consisted of uncontrolled endocrine or cardiac disease, bilateral breast cancer, major surgical procedure within 28 days of initiation of therapy, history of abdominal fistula-gastrointestinal perforation-intra-abdominal abscess, or other malignant diseases. Co-administration of other cancer treatments was not allowed, and hormonal replacement therapy had to be discontinued at least 2 weeks before the initiation of study therapy.

Patients with positive axillary lymph nodes, either by clinical examination or by fine needle aspiration, were treated as having at least N1 disease and were not required to undergo sentinel lymph node biopsy prior to initiation of neoadjuvant therapy. Axillary lymph node dissection was performed in those patients at the time of definitive surgery. Sentinel lymph node biopsy before initiation of neoadjuvant therapy for non-palpable nodes was performed at the discretion of the treating physician. All patients gave informed consent. The protocol was reviewed and approved by each participating institution, and the study followed the Declaration of Helsinki and good clinical practice guidelines.

### Treatment and dose modification

Patients who met the eligibility criteria and signed informed consent were randomly assigned, 2:1, to daily treatment with letrozole 2.5 mg PO and bevacizumab 15 mg/kg IV every 3 weeks (investigational arm, Let/Bev) or daily treatment with letrozole 2.5 mg PO alone (control arm, Let). The duration of each cycle was 3 weeks.

In the absence of treatment delays due to adverse events, treatment continued until one of the following criteria was met: 24 weeks of active therapy or 9 administrations of bevacizumab, disease progression, intercurrent illness that prevented further administration of treatment, any toxicity that resulted in a treatment delay of > 3 weeks, unacceptable grade 3 or 4 non-hematologic adverse event(s), consent withdrawal, and general or specific changes in the patient’s condition that rendered unacceptable for further continuation of treatment in the judgment of the investigator. No dose modifications were allowed for both bevacizumab and letrozole. The maximum allowable length of treatment interruption for bevacizumab was 6 weeks due to the long elimination half-life of the agent. An interval of at least 4 weeks between the last administration of bevacizumab and definitive surgery was required while letrozole was continued until the day before surgery.

### Assessments for safety and efficacy

Patients were monitored for safety and tolerability throughout and after completion of protocol treatment. Toxicity was assessed using the National Cancer Institute Common Terminology Criteria for Adverse Events version 3.0. Physicians’ visits and physical examinations were performed every 6 weeks; laboratory evaluations were performed every 3 weeks in the investigational arm (including a urine protein/creatinine ratio or urine dipstick) and every 6 weeks in the control arm. Study procedures included evaluation of cardiac function at screening and week 24 with echocardiogram or MUGA scan.

At study enrollment, the surgeons were required to state the planned definitive surgery that the patient would have required without preoperative treatment. The type of definitive surgery that the patient underwent after preoperative protocol therapy was recorded.

At 6, 12, ad 18 weeks, patients underwent evaluation of response by physical examination and breast ultrasound. Patients with objective response or stable disease (SD) according to the Response Evaluation Criteria in Solid Tumors (RECIST [16]) continued the same regimen for a maximum of 24 weeks or until progression was observed. Patients with progressive disease (PD) at any time were removed from the protocol and were treated at the investigator’s discretion. At week 24, the evaluation before surgery included a physical evaluation, a breast ultrasound, and a mammogram. Post-surgical adjuvant therapy was administered at the investigator’s discretion.

pCR was defined as the absence of any residual invasive cancer in the resected breast specimen and all sampled ipsilateral lymph nodes (i.e., ypT0/is ypN0) adopting the definition proposed by the FDA [17]; microscopic residual disease was defined as < 5 mm residual invasive cancer in the breast (ypT1mi or ypT1a) and isolated or no tumor cell clusters in the ipsilateral axillary lymph nodes (ypN1mi or ypN0).

Imaging responses were assessed according to RECIST [16] by breast ultrasound or breast MRI in selected cases (whichever modality was consistently obtained throughout the treatment period and most accurately assessed the status of the tumor). Comparisons were made with the same imaging modality in every patient. Pathologic responses were assessed by comparing the maximum cumulative diameter of the target lesion(s) at the time of diagnosis as assessed by imaging studies with the size of the tumor in the final surgical pathology.

### Circulating tumor cells

Levels of circulating tumor cells (CTCs) and circulating endothelial cells (CECs) in the peripheral blood prior to treatment (baseline) and during treatment at weeks 6, 18, and 24 were assessed. The number of CTCs in 7.5 mL of blood was measured using the CellSearch system (Veridex LLC, Raritan, NJ, USA) [18]. CTCs were defined as EPCAM-positive nucleated cells expressing cytokeratins but not the leukocyte common antigen CD45 [19]. In a few cases, e.g., when the available blood volume was below 7.5 mL, we performed CTC enumeration using an EPCAM-based immumomagnetic enrichment and flow cytometry procedure [20]. In this assay, CTCs were defined as nucleated EPCAM-positive and CD45-negative. In order to combine the data from the two assays, we reported the CTC values as CTC per mL of blood.

The number of CECs in 50 μL of blood was measured using a four-color flow cytometric assay performed on FACS Calibur (BD Biosciences, San Jose, CA, USA) [21]. CECs were defined as nucleated, CD34-positive, CD31-positive, and CD45-negative. Other CEC-related populations (CD34-positive and CD45-negative) were also evaluated including (1) nucleated and CD146-positive CECs, (2) activated CECs (CD105-positive and CD31-positive), (3) progenitor CECs (CD133-positive and CD31-positive), and (4) CD146-positive and CD31-positive CECs. CEC values were reported as CEC per microliter of blood.

### Biopsies of primary tumors

Core biopsies (research biopsies) of the primary breast tumor were obtained at baseline and on week 6 after initiation of therapy. Biopsy samples were obtained using a 14–18 gauge core needle. At least three core biopsies were obtained, two snap frozen individually and a third one was taken for the preparation of paraffin-embedded blocks. Frozen tissues were used for high-throughput genomic analyses after macrodissection.

De-identified fresh frozen tumor tissue biopsy specimens were obtained from the University of Alabama at Birmingham’s Comprehensive Cancer Center Tissue Procurement Shared Facility. The specimens were macrodissected by a board-certified pathologist at the Tissue Procurement Shared Facility to enrich for tumor cell content and remove adjacent uninvolved tissue. The dissected specimens were weighed and transferred to a 15-mL conical tube containing ceramic beads, and RLT Buffer (Qiagen) plus 1% BME was added so that the tube contained 35 μL of buffer for each milligram of tissue. The conical tubes containing tissue, ceramic beads, and buffer were agitated in a MP Biomedicals FastPrep machine at 6.5 m/s for 90 s to homogenize the tissue. The homogenized tissue was stored at − 80 °C. Total RNA was extracted from 350 μL of tissue homogenate (equivalent to 10 mg of tissue) using the Norgen Animal Tissue RNA Purification Kit (Norgen Biotek Corporation). Cell lysate was treated with Proteinase K before it was applied to the column and on-column DNAse treatment was performed according to the manufacturer’s instructions. Total RNA was eluted from the columns and quantified using the Qubit RNA Assay Kit and the Qubit 2.0 fluorometer (Invitrogen). We used 70 ng of the purified total RNA for most samples. Small RNA libraries were created as described previously [22] except that no blocking of any small RNA species was performed. In short, small RNA samples had adaptor oligonucleotide ligated to both ends. cDNA was created by reverse transcription and amplified by 15 cycles of PCR. PCR products were purified and run under extreme denaturing conditions on acrylamide gels. Bands corresponding to libraries with insert sizes of 15–30 base pairs were excised and purified. The libraries were sequenced on a HiSeq 2500 (Illumina).

For qPCR assays, 4 μl of purified RNA for each sample was subjected to reverse transcription using the Universal cDNA Synthesis Kit II (Exiqon). Resulting cDNA was diluted 1:20 and combined with primers and 2X Power SYBR mix (ThermoFisher). The primers were obtained from Exiqon (Supplementary Appendix). The qPCR reactions were run on a QuantStudio 6 Flex under default conditions. C_T_ values were called using the QuantStudio software with default settings.

### Statistical considerations

The trial was sized to estimate the within-arm pCR rates to a certain precision; it was not powered to compare arms. Based on a prior pCR rate of 12% in the investigational arm of our pilot trial [15], with *n* = 50, this trial had a standard error of ± 0.052 and, using the Blyth-Still-Casella method, provided a pCR rate and two-sided confidence interval of 5.4–23.3%. In the control arm, with *n* = 25 and projected pCR rate of < 1% [23–25], no pCR events were anticipated and the probability of observing one pCR event was 22%. The purpose of the control arm was to characterize the pCR response as similar to the prior (historical control) trials with single-agent neoadjuvant letrozole.

Descriptive statistics were used to summarize baseline characteristics of all patients by randomization arm in order to assess relative comparability of the two arms of the study. Mean, standard deviation, and range were summarized for continuous variables, and frequencies (percentages) were calculated for categorical variables. The differences between the pCR and response rates were determined along with 95% exact confidence intervals (CIs). All toxicities were recorded and summarized by calculating frequencies and proportions of toxicity grades for each arm.

A Student’s *t* test was used to examine the correlation between response and CTC and CEC values at baseline and at each time point for all patients and patients within each arm. Response was categorized into binary variables, 0 for stable and progressive disease and 1 for partial and complete response while CTC and CEC values were tested as continuous variables. Correlation between response and the changes in CTC and CEC numbers between baseline and other time points and the changes between time points were also examined. Correlations with a *p* value < 0.05 were considered significant.

### Genomic data analyses

Raw sequencing reads were analyzed as described previously [26]. Briefly, reads were aligned to the human whole genome (hg19) requiring perfect matches. Features were created by merging overlapping alignments and total read counts reported for each.

To generate a small RNA-based classifier of treatment response, patients were categorized as responders if they had achieved a pathologic treatment response > 30% and non-responders if they had stable or progressive disease. Pathologic response was assessed by comparing the maximum cumulative diameter of the target lesion(s) at the time of diagnosis as assessed by imaging studies with the size of the tumor in the final surgical pathology. Due to the small number of patients who achieved pCR or microscopic residual disease, a genomic classifier on the basis of achievement of pCR or microscopic residual disease could not be generated. Differential expression of feature counts was assessed using DESeq2 [27]. From previously generated full-length RNA-seq data on these samples (data not presented), we had quality control (QC) metrics (fraction of reads mapping to mRNA and cDNA concentration). Significance was assessed using a likelihood ratio test between the full (response variable + QC metrics) and null (QC metrics) models as implemented in DESeq2.

We predicted the agreement between qPCR and sequencing data using previously described methods [26]. We evaluated the relative proportions of 3′ ends of small RNA features, predicting that those with many, equal proportioned 3′ ends would not yield concordant data between qPCR and sequencing measurements. We then selected small RNA features that had low *p* values from the likelihood ratio test and were predicted to yield concordant qPCR and sequencing measurements. We employed LASSO regression on this subset as implemented in the R package glmnet [28], in order to find small RNA features with optimal ability to classify responder vs. non-responder status. A binomial regression model to predict responder status was created from these optimal classifiers, operating on *z*-score values from both sequencing and qPCR measurements. Raw *C*_T_s from qPCR measurements were normalized using hsa-miR-22-3p. We chose this miRNA based on its robust expression and very low variance across samples in the small RNA sequencing data. Accuracies of model predictions were calculated by summing the true positives and true negatives divided by the total predictions. ROC curve areas and *p* values were calculated using the R package “verification.”

## Results

### Patients and dispositions

Patient demographics and tumor characteristics are shown in Table 1. In the study, 75 patients were randomly assigned, 50 in the Let/Bev arm and 25 in the Let arm (2:1 ratio). All patients received at least 1 cycle of therapy. The median age for the patients enrolled in the Let/Bev arm was 61.4 years (range, 50.4 to 81.9) and 65 for the Let arm (range, 50.4 to 86.3). All patients had an ECOG performance status of 0. The protocol arms were well balanced for race, stage, nodal status, and tumor type. The proportion of patients with grade 2 tumors was higher in the Let arm (76% vs. 58% respectively), while no patient with grade 3 tumors was randomized to the Let arm (16% vs. 0% respectively). Randomization was not stratified for any demographic or disease parameter.

**Table 1:** Patient characteristics

Characteristic	Letrozole alone, *n* = 25	Letrozole/bevacizumab, *n* = 50	*p* value
Age (years)			
Median (range)	65 (50.4–86.3)	61.4 (50.4–81.9)	NSD
Race, *n* (%)			
White	20 (80)	42 (84)	NSD
Black	3 (12)	6 (12)	NSD
Hispanic	1 (4)	2 (4)	NSD
Others	1—Asian (4)	–	
Clinical tumor stage, *n* (%)			
IIA	9 (36)	16 (32)	NSD
IIB	7 (28)	21 (42)	NSD
IIIA	9 (36)	8 (16)	NSD
IIIB	–	5 (10)	NSD
Nodal status, *n* (%)			
N0	9 (36)	21 (42)	NSD
N+	16 (64)	29 (58)	NSD
Tumor type, n (%)			
Invasive ductal carcinoma	16 (64)	29 (58)	NSD
Invasive lobular carcinoma	5 (20)	13* (26)	NSD
Invasive mixed carcinoma	4 (16)	7 (14)	NSD
Others	–	1—mucinous (2)	NSD
Histologic grade, *n* (%)			
Grade I	6 (24)	13 (26)	NSD
Grade II	19 (76)	29 (58)	0.042
Grade III	–	8 (16)	0.025

*Includes one patient with concurrent separate invasive mucinous carcinoma

*NSD* no significant difference

**Fig. 1:**
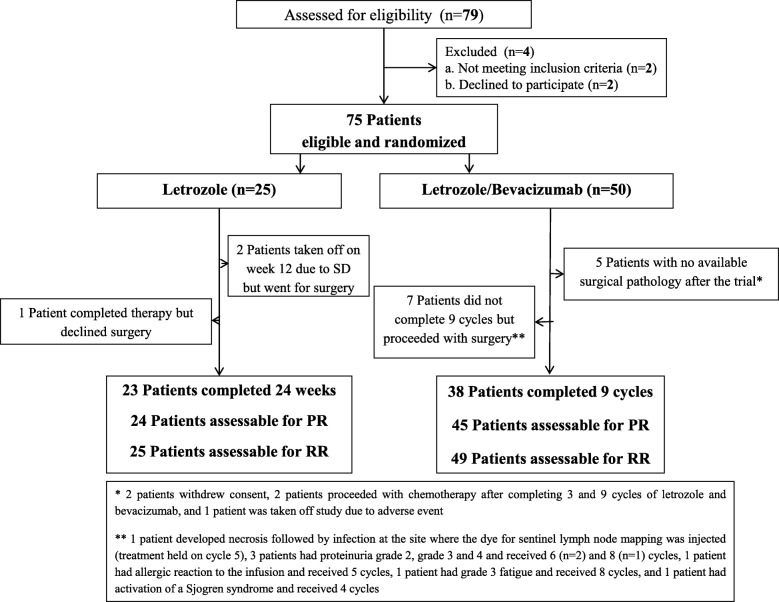
CONSORT diagram

Figure 1 provides the CONSORT diagram for the study. On the Let arm, 92% of the patients (*n* = 24) completed therapy, whereas 76% (*n* = 38) completed therapy on the Let/Bev arm. On the Let arm, discontinuations were due to the absence of response, whereas adverse events were the main reasons for discontinuation in the Let/Bev arm. As specified in the protocol, all patients underwent surgery 4 weeks after the last dose of Bev but continued taking Let until the day before surgery.

### Efficacy

#### Pathologic response

Of the 50 patients enrolled in the Let/Bev arm, 45 patients had available surgical pathology for evaluation of pathologic response (consent withdrawal, *n* = 2; transition to chemotherapy at the discretion of the treating physician, *n* = 2; discontinuation due to toxicity, *n* = 1, Fig. 1). Of the 25 patients in the Let arm, 24 patients had available surgical pathology (1 patient refused surgery upon completion of protocol therapy, Fig. 1).

**Fig. 2:**
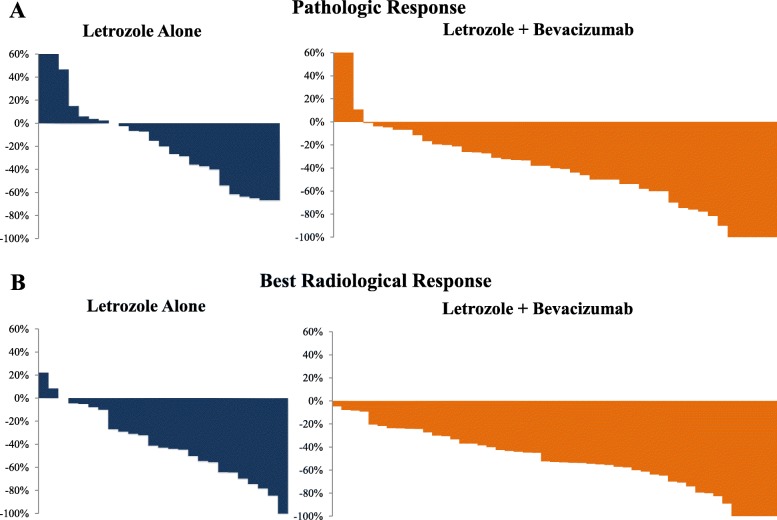
Waterfall plot of response by treatment arm. **a** Pathologic responses were assessed by comparing the maximum cumulative diameter of the target lesion(s) at the time of diagnosis as assessed by imaging studies with the size of the tumor in the final surgical pathology. **b** Radiological responses were assessed according to RECIST by breast ultrasound or breast MRI in selected cases (whichever modality was consistently obtained throughout the protocol therapy and most accurately assessed the status of the tumor)

As seen in Table 2 and Fig. 2a, the rates of pCR, microscopic residual disease, PR, and objective response were numerically higher in the Let/Bev arm vs. Let arm (11.1% vs. none, 8.9%. vs none, 44.4% vs. 37.5%, and 64.4% vs. 37.5% respectively; no formal statistical testing, per protocol). One of the 5 patients who achieved pCR in the Let/Bev arm had residual ductal carcinoma in situ*.* These data are in agreement with the responses reported previously in a single-arm study of letrozole and bevacizumab in the same patient population [15]. The rates of downstaging (from stage 3/2 to stage 1/0) and lymph node reversion (from positive upon diagnosis to negative at the time of surgery) were also numerically higher in the Let/Bev arm (44.4% vs. 37.5% and 40.0% vs. 26.6% respectively). The rates of stable and progressive disease (disease same as or greater than its original radiological assessment at the time of diagnosis) were numerically lower in the Let/Bev arm vs. Let arm (28.8% vs 50% and 6.6% vs 12.5%, respectively). Of the 6 patients with progressive disease in the trial (3 in each arm), the surgical pathology after protocol therapy in 3 patients (Let arm, *n* = 2; Bev/Let arm, *n* = 1) was consistent with invasive lobular carcinoma, grade 1 or 2. Given the diffuse growth pattern of cancer cells and lack of desmoplastic reaction in invasive lobular carcinoma that collectively render its detection by physical examination, imaging, and even gross pathologic evaluation challenging [29], this finding may reflect clinical and radiographic underestimation of the original extent of the disease rather than actual tumor growth..

**Table 2:** Pathologic response per protocol arm

Group	Letrozole, *n* = 24		Letrozole/bevacizumab, *n* = 45	
	*n* (%)	95% CI^†^	*n* (%)	95% CI^†^
Pathologic CR (pCR)	none (0)	0, 14.2%	5^ (11)	3.7%, 24.1%
pCR and microscopic residual disease	none (0)	0, 14.2%	9 (20)	9.6%, 34.7%
pCR and microscopic residual disease—(ITT)*	none (0)	0, 14.2%	9 (18)	8.6%, 31.4%
Partial response	9 (37.5)	18.8%, 59.4%	20 (44.4)	29.6%, 60.0%
Objective response rate	9 (37.5)	18.8%, 59.4%	29 (64.4)	48.8%, 78.1%
Stable disease	12 (50)	29.1%, 70.9%	13 (28.8)	16.4%, 44.3%
Progressive disease	3 (12.5)	2.7, 32.4%	3 (6.6)	1.4%, 18.3%
Downstage (3/2 to 0/1)	9 (37.5)	18.8%, 59.4%	20 (44.4)	29.6%, 60.0%
Node reversion (positive to negative)	4/15 (26.6)	7.8%, 55.1%	10/25 (40)	21.1%, 61.3%

^†^Exact (Clopper-Pearson) confidence interval

^One of the 5 patients who achieved a pCR had residual ductal carcinoma in situ

*ITT (intention to treat population): this analysis assumes that none of the patients who did not undergo surgery (letrozole group, *n* = 1; letrozole/bevacizumab group, *n* = 5) achieved a pCR or microscopic residual disease

pCR and microscopic residual disease were seen only in patients with grade 1 or 2 disease at diagnosis. Additionally, all patients who achieved pCR and microscopic residual disease had stage 2 disease at diagnosis.

Of the 45 patients assigned to the Let/Bev arm with available surgical pathology, 37 were deemed at diagnosis to require mastectomy. Of these patients, 15 (40.5%; 95% CI, 24.8–57.9%) underwent lumpectomy following preoperative letrozole and bevacizumab. Of the 23 patients assigned to the Let arm with available surgical pathology (declined surgery *n* = 1, definitive surgery without preoperative medical therapy not assessed *n* = 1), 17 were deemed at diagnosis to require mastectomy. Of these patients, 9 (53%; 95% CI, 27.8–77%) underwent lumpectomy following preoperative letrozole alone.

#### Radiological response

Forty-nine patients in the Let/Bev arm (one patient withdrew consent before the first assessment of radiological response due to non-protocol related issues) and 25 patients in the Let arm were evaluable for radiological response according to RECIST [16]; results are summarized in Table 3 and Fig. 2b. Complete, partial, and objective radiographic response rates were numerically higher in the Let/Bev arm (10% vs. 4%, 69% vs. 64%, and 79% vs. 68% respectively). Accordingly, the rates of stable and progressive disease were numerically lower in the Let/Bev arm vs. Let arm (21% vs. 28% and none vs. 4%, respectively). The absolute mean percent reduction of the maximum tumor diameter by imaging was − 51.08% (95% CI, − 43.5 to − 58.6) in the Let/Bev arm vs. − 39.2% in the Let arm (95% CI, − 26.4 to − 52.2). Two patients in the Let arm and 3 in the Let/Bev arm discontinued treatment due to inadequate clinical response by physical evaluation and imaging; both patients in the Let arm went for surgery without additional neoadjuvant therapy while in the Let/Bev arm, 2 patients proceeded to chemotherapy after completion of cycles 3 and 9 and 1 patient withdrew consent.

**Table 3:** Best radiological response per protocol arm

Best radiological responses	Letrozole, *n* = 25		Letrozole/bevacizumab, *n* = 49	
	*n* (%)	95% CI^†^	*n* (%)	95% CI^†^
Complete response (%) (no evidence of residual tumor)	1 (4)	0.1%, 20.4%	5 (10)	3.4%, 22.2%
Partial response	16 (64)	42.5%, 82.0%	34 (69)	54.6%, 81.7%
Objective response rate	17 (68)	46.5%, 85.1%	39 (79)	65.7%, 89.8%
Stable disease	7 (28)	12.1%, 49.4%	10 (21)	10.2%, 34.3%
Progressive disease	1 (4)	0.1%, 20.4%	0 (0)	0, 7.3%
Mean tumor maximum diameter change	− 39%	− 26.4, − 52.2	− 51%	− 43.5, − 58.6

^†^Exact (Clopper-Pearson) confidence interval

### Safety

Twenty-five patients in the Let arm and 50 patients in the Let/Bev arm received at least one cycle of therapy and were eligible for toxicity evaluation. The majority of adverse events were grades 1 and 2.

As summarized in Table 4, the most common adverse events observed in more than 5% of the patients in the Let arm deemed by the investigators to be possibly related to the aromatase inhibitor were hot flashes (28%), arthralgias/joint stiffness (24%), fatigue (16%), myalgias/cramps (12%), nausea/vomiting (8%), and night sweats (8%). No grade 4 toxicity was seen in the Let arm and only 2 patients (8%) had grade 3 arthralgias/joint stiffness. No patient discontinued the medication in the letrozole alone arm due to toxicity.

**Table 4:** Adverse events of any grade seen in > 5% of patients in either protocol arm

	Letrozole, *n* = 25		Letrozole/Bevacizumab, *n* = 50	
	Number of patients (percent)			
Adverse events	Any grade	Grade ≥ 3	Any grade	Grade ≥ 3
Hot flashes	7 (28)	0	9 (18)	0
Arthralgia/joint stiffness	6 (24)	2 (8)	13 (26)	2 (4)
Fatigue	4 (16)	0	7 (14)	1 (2)
Myalgias/cramps	3 (12)	0	3 (6)	0
Nausea/vomiting	2 (8)	0	1 (2)	0
Night sweats	2 (8)	0	2 (4)	0
Mood swings/anxiety/agitation	1 (4)	0	3 (6)	0
Vaginal dryness	1 (4)	0	3 (6)	0
Hypertension	1 (4)	0	16 (32)	4 (8)
Hemorrhagic and thrombotic events	0	0	8 (16)	3 (6)
Headache	1 (4)	0	7 (14)	0
Proteinuria	0	0	5 (10)	2 (4)
Dyspnea	1 (4)	0	5 (10)	0
Skin (rash, discoloration, pruritus)	2 (8)	0	4 (8)	0
Lower extremity edema	0	0	3 (6)	0

Adverse events known to be associated with letrozole were observed in similar frequencies in both arms. As expected, there was additive toxicity in the Let/Bev arm as adverse events known to be associated with bevacizumab were observed. The most common adverse events observed in more than 5% of the patients in the Let/Bev arm deemed by the investigators to be possibly related to the protocol agents were arthralgias/joint stiffness (26%), hot flashes (18%), fatigue (14%), myalgias/cramps (6%) possibly attributable to letrozole; hypertension (32%), hemorrhagic and thrombotic events (16%, includes epistaxis (grade 1, *n* = 1; grade 2, *n* = 1), bleeding gingivae (grade 1, *n* = 1), hematoma (grade 1, *n* = 1), rectal bleeding (grade 1, *n* = 1), cerebrovascular ischemia (grade 3, *n* = 1), hematemesis (grade 3, *n* = 1), and syncope (grade 3, *n* = 1)), headache (14%), proteinuria (10%), dyspnea (10%), and rash (8%), possibly attributable to bevacizumab. The most frequent grade 3 adverse events seen in the Let/Bev arm were hypertension (8%), arthralgias/joint stiffness (4%), and proteinuria (2%). Only 1 patient had grade 4 toxicity in the combination arm which was proteinuria (2%). Seven patients discontinued therapy in the Let/Bev arm due to adverse events, but pathology was available after the surgical procedure: 1 patient developed necrosis followed by infection at the site where the dye for sentinel lymph node mapping was injected (protocol therapy discontinued on cycle 5); 3 patients had proteinuria (grades 2, 3, and 4, *n* = 1 each) and received 6, 6, and 8 cycles, respectively; 1 patient had allergic reaction to the infusion and received 5 cycles; 1 patient had grade 3 fatigue and received 8 cycles; and 1 patient had activation of a Sjögren syndrome and received 4 cycles. In addition, one patient discontinued therapy in the Let/Bev arm due to grade 3 cerebrovascular ischemia but surgical pathology was not available. No healing problems at the time of surgery were seen.

Three serious adverse events were reported in the Let/Bev arm which were classified by the investigators as possibly related to the protocol therapy: 1 patient was admitted with confusion to the hospital due to a possible TIA (patient continued therapy), 1 patient had a neck abscess which required admission to the hospital for IV antibiotics, and 1 patient had grade 3 cerebrovascular ischemia (patient was taken off study). This last patient had presented with expressive aphasia. Thorough workup did not reveal acute infarct, mass, or intracranial hemorrhage. Echocardiogram was consistent with moderate to severe aortic stenosis. Her hospital stay was extended to provide adequate blood pressure control (blood pressure was 220/90 mmHg at presentation). On follow-up, there were no residual neurologic symptoms. No deaths associated with the treatment agents were seen in the trial.

### Circulating tumor cells and circulating endothelial cells

CTCs were detected only in 14% of the patients. The mean number of circulating tumor cells was 0.05/mL (SD 0.24, range 0–1.7). Due to the limited number of patients with CTCs, the numbers at baseline and other time points as well as the change in CTCs between time points did not show a statistically significant correlation with response (data not shown).

Samples for CEC analysis at baseline were available in 77% of the patients (Supplementary Table 1). At baseline, the mean number of CD31+ CECs was 20.6 cells/μL (standard deviation [SD] 63.0, range 2.7–489.3); CD146+ CECs, 2.9 cells/μL (SD 3.2, range 0–13.9); activated CECs (CD105+/CD31+cells), 12.9 cells/μL (SD 44.4, range 1.2–296.2); progenitor CECs (CD133+/CD31+), 0.8 cell/μL (SD 1.6, range 0–9.7); and CD146+/CD31+ CECs, 0.7 cell/μL (SD 2.5, range 0–18.5). Absolute numbers and changes in CEC levels did not show significant correlation with response when all patients, regardless of the treatment arm in which they were enrolled, were considered (Supplementary figure 1). However, in the Let/Bev arm, we observed a statistically significant correlation between response and the levels of activated CECs (CD105+/CD31+) at week 18 (*p* = 0.0432). In addition, changes in the levels of activated CECs (CD105+/CD31+) (*p* = 0.0038), progenitor CECs (CD133+/CD31+) (*p* = 0.0120), and CD146+/CD31+ CECs (*p* = 0.0087) between weeks 6 and 18 significantly correlated to response.

### Generation of a small RNA classifier that predicts response to letrozole plus bevacizumab

**Fig. 3:**
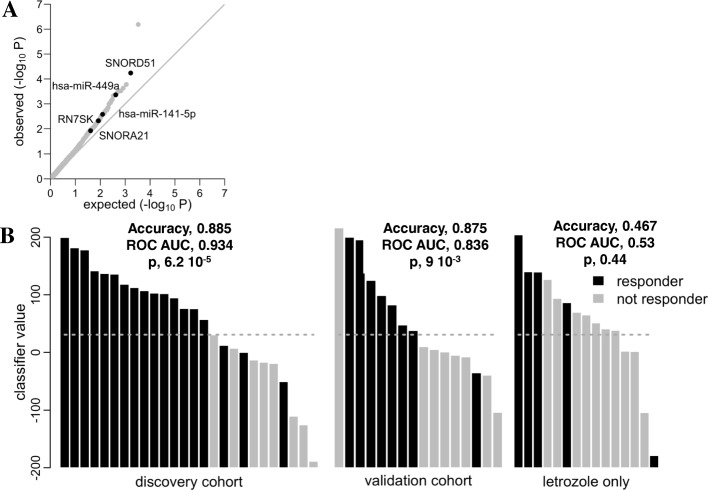
**a** Quantile-quantile plot of small RNA *p* values for association with letrozole/bevacizumab response. The observed *p* values from the association of small RNA expression values with letrozole/bevacizumab response (see the “Methods” section) are plotted against the null (uniform) distribution. Small RNAs selected by LASSO regression for an optimal classifier model (see the “Methods” section) are marked by black dots and labeled. **b** Classifier model performance on three cohorts. Classifier values calculated from qPCR data for each patient tumor sample (*x*-axis) across three cohorts are plotted on the *y*-axis (see the “Methods” section). Bar color indicates responder status. The dotted line indicates the common threshold used to calculate model accuracy. The “discovery cohort” is the cohort of patients who received letrozole/bevacizumab in the present study. The “validation cohort” is the cohort of patients who received the same treatment in a separate independent clinical trial [15]. The “letrozole cohort” consists of patients who received letrozole in the present study. The model was trained on sequencing data from the discovery cohort

Macrodissected tumor tissue from research biopsies obtained at the time of diagnosis was successfully sequenced in 26 and 15 patients assigned to Let/Bev and Let arms respectively. Differential gene expression analyses yielded a large number of small RNAs to be significantly associated with response to letrozole/bevacizumab. hsa-mIR-187-3p was the highest ranking differentially expressed small RNA between responders and non-responders to Let/Bev in terms of significance (Supplementary Appendix). Increased hsa-mIR-187-3p expression in breast cancer has been associated with an aggressive, invasive phenotype and has been shown to be an independent predictor of outcome [30]. To overcome the biases associated with small RNA library preparations and the complexities of genomic data analyses, we filtered for small RNAs likely to reproduce the sequencing data when measured by qPCR (see the “Methods” section). We identified a set of five small RNAs as an optimal letrozole/bevacizumab classifier by LASSO regression (Fig. 3a). These include two miRNAs (hsa-miR-141-5p, hsa-miR-449), two small nucleolar RNAs (SNORD51, SNORA21), and fragments of 7SK RNA. hsa-mIR-187-3p was not predicted to generate qPCR data concordant with sequencing measurements.

To confirm the sequencing data for these five candidate classifier small RNAs, we measured them in the same set of samples by qPCR. We found a significant agreement (*p* < 0.01) between sequencing and qPCR data for all but one (SNORA21, Supplementary Figure 2). To evaluate their performance as classifiers of response to letrozole/bevacizumab in both types of measurement (sequencing and qPCR), we *z*-scored the sequencing data and created a binomial regression model comprising the five LASSO-selected small RNAs. When we applied the classifier to the sequencing data that it was trained on, the classifier had perfect performance (ROC AUC = 1, *p* = 7.1 × 10^−6^). We then applied the classifier to *z*-scored qPCR data from the same training samples and observed that the classifier still performed well (ROC AUC = 0.934, *p* = 6.2 10^−5^, accuracy = 0.885; Fig. 3b). We chose a threshold for calling responders in this data set that maximized model accuracy (Fig. 3b).

As a robust verification of the classifier, we applied it to *z*-scored qPCR data from breast tumor samples collected from patients that received the same treatment (letrozole/bevacizumab) in a separate independent clinical trial [15]. We successfully sequenced 16 macrodissected tumor samples from the validation cohort. We observed good performance of the classifier in these samples as well (Fig. 3b), with only moderate decrease in performance metrics (ROC AUC = 0.836, *p* = 9.0 × 10^−3^, accuracy = 0.875). Lastly, we applied the model to patients from the main trial that received letrozole alone to evaluate the specificity of the classifier for the letrozole/bevacizumab combination, rather than for letrozole alone. We observed non-significant performance of the classifier on patients who received letrozole alone (ROC AUC = 0.53, *p* = 0.44, accuracy = 0.467; Fig. 3b).

## Discussion

The addition of bevacizumab to letrozole capitalizes on multiple lines of evidence that implicate VEGF and angiogenesis as major mediators of resistance to endocrine therapies in luminal breast cancer [2–6, 13–15]. This study met its primary endpoint by determining the pCR rate of neoadjuvant/preoperative letrozole in combination with bevacizumab for 24 weeks to be 11.1% (CI, 3.7–24.1%) (20% including pCR rates and rates of microscopic residual disease). This pCR rate is highly similar to the 12% observed in our pilot study conducted in the same patient population [15] and at least comparable to the pCR rate achieved with neoadjuvant anthracycline-taxane-based chemotherapy [31]. To our knowledge, this pCR rate is the highest reported in endocrine-based neoadjuvant clinical trials, exceeding the < 10% pCR rate reported in other preoperative studies combining endocrine therapy with growth factor pathway inhibitors [32]. In neoadjuvant trials that compared letrozole with or without taselisib and with or without everolimus, the pCR rate in the combination arm was 1.8% [25] and 1.4% [23], respectively. Similarly, the pCR rates of preoperative combinations of endocrine therapy with CDK4/6 inhibitors have been low (0% [33] and 3.8% [34]) as well. No pCR or microscopic residual disease was seen in the control arm, and this result is consistent with multiple prior trials with preoperative aromatase inhibitors. It should be noted that our study was not powered for direct comparisons between arms and a larger study will be required to further confirm these results.

Interestingly, pCRs were confined to patients with stage 2 and well to moderately differentiated tumors. The presence of established angiogenesis in larger tumors promoted and sustained by multiple growth factors may underpin this observation. Indeed, in preclinical studies, anti-VEGF therapies have been shown to preferentially target smaller peripheral blood vessels while centrally located large vessels were spared [35].

Although the toxicities in the investigational arm were not synergistic, the addition of bevacizumab did result in a set of adverse events, known to be associated with bevacizumab, including hypertension, proteinuria, and hemorrhagic and thrombotic events. The nature and frequencies of the bevacizumab-associated adverse events in this study were very similar to the ones reported in other studies of bevacizumab combined with endocrine therapy [36–38]. We should acknowledge the uniformly very good performance status at baseline, generally favorable prognosis, and absence of residual toxicities from prior therapies in this population as well as the favorable toxicity profile of letrozole.

The achievement of pCR with preoperative therapy has been associated with favorable long-term outcomes, and the addition of bevacizumab to preoperative chemotherapy has led to higher pCR rates [31, 39–41]. Notably though, this benefit has not consistently translated into a long-term disease-free or definitive overall survival advantage [42–45]. A limitation of our study is the lack of long-term follow-up for recurrence, survival, and toxicities associated with bevacizumab. However, the choice of postoperative therapy was left to the physician’s discretion and many patients with large residual disease or limited treatment effect eventually received adjuvant cytotoxic chemotherapy. Accordingly, whether the higher pCR rate and better overall responses achieved with the addition of bevacizumab translate into better long-term outcomes remains an open question. The controversies regarding antiangiogenic therapy underscore the need to identify a biomarker for optimal patient selection; such a biomarker has remained largely elusive [46–48]. To address this gap in the knowledge, in the present study, we explored the utility of circulating tumor and endothelial cells and of a small RNA-based genomic classifier.

The level of CTCs in these patients with early-stage hormone receptor-positive breast cancer was low, in agreement with the literature [49]. The number of CTCs before and during treatment and the changes in levels during therapy were not associated with response. However, the low number of patients with detectable CTCs limits meaningful conclusions. In contrast, CECs were detected in most patients. Interestingly, we observed a correlation between response to letrozole and bevacizumab and the levels of activated CECs at week 18. Changes in the levels of certain CEC subpopulations (activated CECs, progenitor CECs, and CD146+/CD31+ CECs) between weeks 6 and 18 were also significantly associated with response to letrozole and bevacizumab. The significance of these associations remains unclear considering the lack of any correlations at other time points and the limited sample size.

By contrast to the CTCs and CECs, our small RNA-based classifier may constitute a potential biomarker predictive of response to bevacizumab. All small RNA components of the classifier have been implicated in the pathobiology of breast cancer. The hsa-miR-141-5p is the less abundant product from the same pre-miRNA with hsa-miR-141-3p and may in fact represent a surrogate measurement of hsa-miR-141-3p. Overexpression of hsa-miR-141-3p and its family member hsa-miR-200c-3p in MDA-MB-231 breast cancer cells promotes significant migration and invasion and enhances VEGF-A secretion [50]. VEGF-A neutralizing antibodies abrogate these phenotypic consequences conferred by hsa-miR-141-3p and hsa-miR-200c-3p overexpression [50]. hsa-miR-449a targets cysteine-rich intestinal protein 2 (CRIP2) mRNA, a transcription factor and a tumor suppressor [51]. CRIP2 interacts with the NF-κB/p65 to inhibit its DNA-binding ability to the promoter regions of the major pro-angiogenesis cytokines critical for tumor progression, including VEGF [51]. Indeed, increased expression of CRIP2 is associated with impaired tumor angiogenesis [52]. C/D box snoRNAs (like small nucleolar RNA, C/D box 51 (SNORD51)) and H/ACA box snoRNAs (like small nucleolar RNA, H/ACA box 21(SNORA21)) define the target sites for 2′-O-ribose methylation and pseudouridylation on ribosomal RNA (rRNA), respectively [53]. To our knowledge, a direct association between small nucleolar RNAs and VEGFA expression or function has not been reported. The possibility exists though that altered rRNA modifications introduced by the small nucleolar RNAs may modulate the RNA affinities and translational capabilities of the ribosomes and, consequently, prioritize the translation of VEGF mRNA as shown with the rRNA methyl-transferase fibrillarin [54, 55]. Lastly, 7SK snRNA and the La-related protein LARP7 are required for the integrity of the 7SK snRNP complex [56]. This complex sequesters the positive transcription elongation factor b (P-TEFb). Decreased levels of LARP7 and 7SK snRNA redistribute P-TEFb to the transcriptionally active super elongation complex, resulting in accelerated transcription of transcription factors that promote breast cancer epithelial-mesenchymal transition, invasion, and metastasis [56]. Although a direct association between 7SK snRNA or 7SK snRNP and VEGFA expression or function has not been reported, the possibility exists that low levels of 7SK snRNA may allow for upregulated VEGFA transcription. Although our small RNA-based classifier was verified in an independent cohort, incorporation of the classifier as a correlative study in clinical trials with bevacizumab will allow for further refinements and validation. We should note that we also performed RNA-seq on available tumor samples but RNA-seq data analyses did not yield significant classifiers.

## Conclusion

In stage 2 and 3 hormone receptor-positive and Her2-negative breast cancer, the achievement of pCR with endocrine therapy combinations, including combinations with PI3K inhibitors and CDK4/6 inhibitors has been an elusive target. In this patient population, the addition of bevacizumab to preoperative letrozole resulted in a pCR rate of 11.1% (95% CI, 3.7–24.1%) at the cost of additive toxicities. We have developed a small RNA-based classifier, which, on the basis of the levels of five small RNAs in a pretreatment biopsy, can select patients more likely to respond to bevacizumab.

## Supplementary information

**Additional file 1.** Supplementary Figure 1 Changes in circulating endothelial cells (CECs) during protocol therapy. Enumeration at baseline and at weeks 6, 18, and 24 of circulating endothelial cells (CECs) and related populations in blood samples collected from all patients assigned in both protocol arms. Absolute numbers and changes in CEC levels did not significantly correlate with response.

**Additional file 2 **Supplementary Figure 2 Comparison of small RNA sequencing data to qPCR. Upper panels. For the 5 small RNAs selected as the optimal classifier by LASSO, we plotted sequencing values (“vsd” for variance stabilized data by DESeq2, x-axis) versus qPCR data (-dCt, y-axis). The R-squared and *p*-value of the goodness-of-fit by linear regression are provided for each small RNA. Lower panels. Boxplots for each measurement type (qPCR: “-dCt”; sequencing: “vsd”) for each small RNA selected as the optimal classifier by LASSO between non-responders and responders to letrozole/bevacizumab. The *p*-values are from linear regression for qPCR data (“-dCt” y-axis) and from DESeq2 (“vsd” y-axis) for sequencing data.

**Additional file 3.** Supplementary Table 1 CEC enumeration in all trial participants.

**Additional file 4.** Appendix 1. LASSO selected classifiers and qPCR assay information.

**Additional file 5.** Appendix 2. Summary statistics for all tested small RNA features in sequencing data (sorted by letozole/bevacizumab responder P).

## Data Availability

All raw and processed sequencing data generated in this study have been submitted to the NCBI Gene Expression Omnibus (GEO; http://www.ncbi.nlm.nih.gov/geo/) under accession number GSE142308. Analyzed data and the R code used to generate the main and supplementary figures are included as supplementary information files.

## Abbreviations

CECs: Circulating endothelial cells
CI: Confidence interval
CR: Complete response
CTCs: Circulating tumor cells
ER: Estrogen receptor
MRI: Magnetic resonance imaging
MUGA: Multigated acquisition
pCR: Pathologic complete remission
PD: Progressive disease
PR: Partial response
RECIST: Response Evaluation Criteria in Solid Tumors
SD: Stable disease
VEGF: Vascular endothelial growth factor
