# Supplementary material for: TBCRC 002: a phase II, randomized, open-label trial of preoperative letrozole with or without bevacizumab in postmenopausal women with newly diagnosed stage 2/3 hormone receptor-positive and HER2-negative breast cancer
Source: Breast Cancer Res. 2020 Feb 18;22:22. doi: 10.1186/s13058-020-01258-x (PMC7027068; doi:10.1186/s13058-020-01258-x)
Supplement: Supplementary file 1 — Additional file 1. Supplementary Figure 1 Changes in circulating endothelial cells (CECs) during protocol therapy. Enumeration at baseline and at weeks 6, 18, and 24 of circulating endothelial cells (CECs) and related populations in blood samples collected from all patients assigned in both protocol arms. Absolute numbers and changes in CEC levels did not significantly correlate with response. [file 13058_2020_1258_MOESM1_ESM.pptx]

## Slide 1
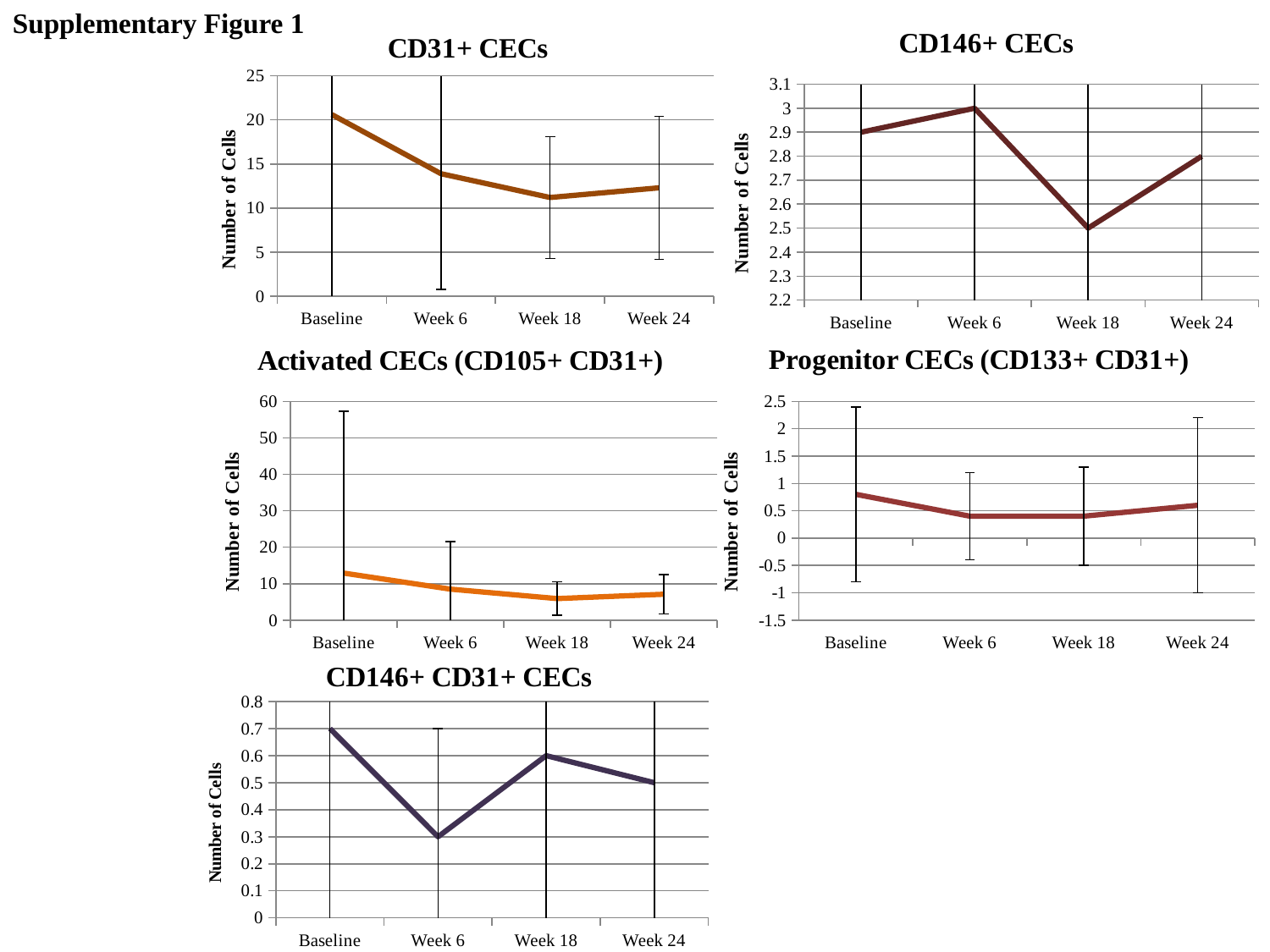

Supplementary Figure 1
### Chart: CD146+ CECs
| Category | Mean |
|---|---|
| Baseline | 2.9 |
| Week 6 | 3.0 |
| Week 18 | 2.5 |
| Week 24 | 2.8 |
### Chart: CD31+ CECs
| Category | Mean |
|---|---|
| Baseline | 20.6 |
| Week 6 | 13.9 |
| Week 18 | 11.2 |
| Week 24 | 12.3 |
### Chart: Progenitor CECs (CD133+ CD31+)
| Category | Mean |
|---|---|
| Baseline | 0.8 |
| Week 6 | 0.4 |
| Week 18 | 0.4 |
| Week 24 | 0.6 |
### Chart: Activated CECs (CD105+ CD31+)
| Category | Mean |
|---|---|
| Baseline | 12.9 |
| Week 6 | 8.5 |
| Week 18 | 5.9 |
| Week 24 | 7.1 |
### Chart: CD146+ CD31+ CECs
| Category | Mean |
|---|---|
| Baseline | 0.7 |
| Week 6 | 0.3 |
| Week 18 | 0.6 |
| Week 24 | 0.5 |
