# Supplementary material for: TBCRC 002: a phase II, randomized, open-label trial of preoperative letrozole with or without bevacizumab in postmenopausal women with newly diagnosed stage 2/3 hormone receptor-positive and HER2-negative breast cancer
Source: Breast Cancer Res. 2020 Feb 18;22:22. doi: 10.1186/s13058-020-01258-x (PMC7027068; doi:10.1186/s13058-020-01258-x)
Supplement: Supplementary file 2 — Additional file 2 Supplementary Figure 2 Comparison of small RNA sequencing data to qPCR. Upper panels. For the 5 small RNAs selected as the optimal classifier by LASSO, we plotted sequencing values (“vsd” for variance stabilized data by DESeq2, x-axis) versus qPCR data (-dCt, y-axis). The R-squared and p-value of the goodness-of-fit by linear regression are provided for each small RNA. Lower panels. Boxplots for each measurement type (qPCR: “-dCt”; sequencing: “vsd”) for each small RNA selected as the optimal classifier by LASSO between non-responders and responders to letrozole/bevacizumab. The p-values are from linear regression for qPCR data (“-dCt” y-axis) and from DESeq2 (“vsd” y-axis) for sequencing data. [file 13058_2020_1258_MOESM2_ESM.pptx]

## Slide 1
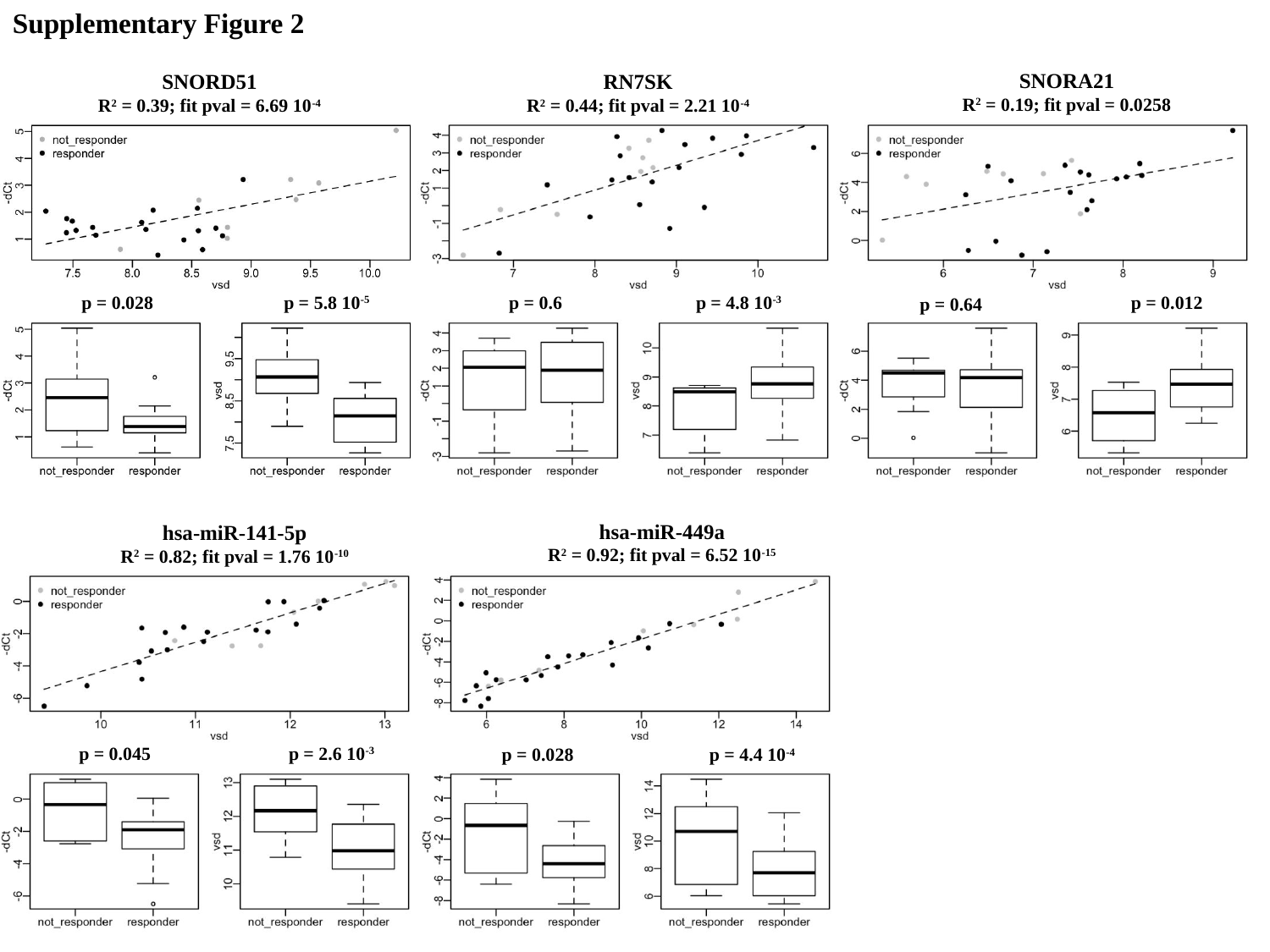

Supplementary Figure 2
SNORA21
R2 = 0.19; fit pval = 0.0258
SNORD51
R2 = 0.39; fit pval = 6.69 10-4
RN7SK
R2 = 0.44; fit pval = 2.21 10-4
p = 0.028
p = 5.8 10-5
p = 0.6
p = 0.012
p = 4.8 10-3
p = 0.64
hsa-miR-449a
R2 = 0.92; fit pval = 6.52 10-15
hsa-miR-141-5p
R2 = 0.82; fit pval = 1.76 10-10
p = 0.045
p = 2.6 10-3
p = 0.028
p = 4.4 10-4
