# Supplementary material for: TBCRC 002: a phase II, randomized, open-label trial of preoperative letrozole with or without bevacizumab in postmenopausal women with newly diagnosed stage 2/3 hormone receptor-positive and HER2-negative breast cancer
Source: Breast Cancer Res. 2020 Feb 18;22:22. doi: 10.1186/s13058-020-01258-x (PMC7027068; doi:10.1186/s13058-020-01258-x)
Supplement: Supplementary file 3 — Additional file 3. Supplementary Table 1 CEC enumeration in all trial participants. [file 13058_2020_1258_MOESM3_ESM.docx]

**Supplementary Table 1.** CEC enumeration in all trial participants.

| Cell population | Time Point | N* | Mean | SD** | Median | Minimum | Maximum |
| --- | --- | --- | --- | --- | --- | --- | --- |
|  |  |  |  |  |  |  |  |
| CECs ( CD31+) | Baseline | 58 | 20.6 | 63.0 | 11.0 | 2.7 | 489.3 |
|  | Week 6 | 64 | 13.9 | 13.1 | 11.5 | 0.3 | 84.7 |
|  | Week 18 | 54 | 11.2 | 6.9 | 9.6 | 1.4 | 31.6 |
|  | Week 24 | 57 | 12.3 | 8.1 | 10.9 | 0.9 | 42.5 |
|  |  |  |  |  |  |  |  |
| CECs (CD146+) | Baseline | 59 | 2.9 | 3.2 | 2.1 | 0.0 | 13.9 |
|  | Week 6 | 62 | 3.0 | 4.3 | 1.6 | 0.0 | 23.8 |
|  | Week 18 | 54 | 2.5 | 2.9 | 1.6 | 0.0 | 15.3 |
|  | Week 24 | 55 | 2.8 | 4.0 | 1.1 | 0.0 | 16.5 |
|  |  |  |  |  |  |  |  |
| Activated CECs | Baseline | 43 | 12.9 | 44.4 | 4.9 | 1.2 | 296.2 |
| (CD105+,CD31+) | Week 6 | 41 | 8.5 | 13.1 | 5.3 | 0.1 | 69.2 |
|  | Week 18 | 30 | 5.9 | 4.6 | 5.0 | 1.4 | 20.0 |
|  | Week 24 | 33 | 7.1 | 5.4 | 5.9 | 0.7 | 25.6 |
|  |  |  |  |  |  |  |  |
| Progenitor CECs | Baseline | 54 | 0.8 | 1.6 | 0.2 | 0.0 | 9.7 |
| (CD133+, CD31+) | Week 6 | 59 | 0.4 | 0.8 | 0.1 | 0.0 | 4.8 |
|  | Week 18 | 51 | 0.4 | 0.9 | 0.1 | 0.0 | 5.0 |
|  | Week 24 | 55 | 0.6 | 1.6 | 0.2 | 0.0 | 10.8 |
|  |  |  |  |  |  |  |  |
| CECs | Baseline | 56 | 0.7 | 2.5 | 0.1 | 0.0 | 18.5 |
| (CD146+, CD31+) | Week 6 | 60 | 0.3 | 0.4 | 0.0 | 0.0 | 1.7 |
|  | Week 18 | 52 | 0.6 | 2.4 | 0.0 | 0.0 | 17.0 |
|  | Week 24 | 54 | 0.5 | 2.6 | 0.0 | 0.0 | 19.1 |

***N**, number of patients who submitted samples

****SD**, standard deviation
